# Royal National Hospital for Consumption, Ventnor

**Published:** 1902-03-22

**Authors:** 


### ROYAL NATIONAL HOSPITAL FOR CONSUMPTION, VENTNOR.

LORD ROSEBERY IN THE CHAIR.

If any hopes had been entertained by the governors, as they certainly were by the unusually large press contingent, that Lord Rosebery would make a "pronouncement" on consumption, on Thursday, the 13th inst., they were doomed to be disappointed. The president confined his remarks chiefly to two points: the method of admission, and the need for extension.

The meeting, which was the annual general meeting of governors, was held at the Alpine Club Hall, Savile Row, London, and by a few minutes to five the room, seating perhaps some hundred and twenty persons, was full, many ladies being present. There was a flutter of excitement, and a good deal of applause, when, at five o'clock, Lord Rosebery entered from the Committee Room, and took his place on the platform, requesting the secretary to read some of the report. With him on the platform were Mr. G. T. Powell, deputy-chairman; the treasurer, Mr. George W. Dawes; the Right Hon. Lord Alverstone, G.C.M.G, Lord Chief Justice of England; Dr. Robert Robertson, and Mr. Morgan, secretary. Other honorary officers were scattered among the audience.

Privileges of Subscribers.

Lord Rosebery, whose rising was the signal for more applause, said it gave him great pleasure to move the adoption of the report. It contained one or two personal features of considerable interest: they had to mourn the loss of their patroness the late Queen Victoria, and to welcome her successor, who had deigned to fill her place as patron. They could not forget the deep interest the King had shown in all matters relating to consumption, its cause and cure. Then they had lost the services of their excellent and energetic chairman, Lord Alverstone, whose work as Lord Chief Justice prevented his continuing in that office, though he would still have a place on the Board of Management. There was one point which seemed to him of very considerable importance—viz. the privileges of subscribers. It was proposed to modify these as regarded the letters of introduction. This had become necessary owing to the success of the hospital. At present a patient had to wait about 20 weeks between his acceptance and his admission at Ventnor, and an interval of five months must necessarily mean something very grave indeed to the patient. Personally, the speaker had long had this matter under consideration, and had thought of bringing it before the committee. As President, he received a large number of letters from patients who imagined he had a store of letters to give away, and also from those who, having obtained letters, thought he had some secret power of accelerating the admission of the case. The accommodation was limited, and it was better that there should not be this strain upon it. If the qualifications of a Governor were raised, more money would be obtained, and with more money they could increase the accommodation, and therefore the means of meeting the strain. He supposed there was no one in that room who was not alive to the claims of consumption. The accommodation, as he had said, was of a very limited character; the number of patients treated last year was 923, or 24 more than in the preceding year. "We would gladly treat more than 923 patients," he continued, "which is the maximum number the hospital can take; for there are no less than a quarter of a million persons in Great Britain who are suffering from consumption. When you consider the vast gap between a quarter of a million and the number we can accommodate, you will see the need we have to increase the scope of the hospital. Our general meeting does not ordinarily call for a great deal of attention from the press, but we are anxious to put forward an appeal for larger means for dealing with this frightful curse to humanity. The subject is in the air; people are alive to it; the recent Congress on Tuberculosis did much to ventilate the subject, and I think the time is ripe for asking the public to give this hospital larger support than they have done in the past. That is why I am here to-day. I am quite aware that I am not so great a working bee as my learned friend on my left (Lord Alverstone), but even he does not yield to me in the interest I take in the matter. My relations on my wife's side were deeply interested in it, and they contributed largely to it; and it is largely for this reason—though I have others—that I feel such deep interest in it myself. As I cannot add to the telling force of the figures I have quoted, I move the adoption of the report."

The Deputy-chairman, Mr. George Thompson Powell, in seconding the motion, congratulated the meeting on having Lord Rosebery there, and himself on having been clever enough to capture him. He then proceeded to give

A Few Statistics.

People often asked what good was really done, if any, by the treatment of consumption. The cases at Ventnor were divided into "complete" and "incomplete," the incomplete cases being those in the hospital on the last day of December, and who had not had a fair chance. It was best, said Mr. Powell, to deal with completed cases, and of these there were 776 last year. Of this number, 203 left very much improved, 171 were much improved, 262 were improved, 95 remained *in statu quo*, 21 were worse, and 24 died. By "very much improved," the doctors meant that the disease was arrested for a time. The gross average for the past four or five years was 865, and of these 183 were very much improved, and 400 much improved, *i.e.* nearly 80 per cent. of the whole number. The rest got worse and died. Considering the difficulties under which the hospital laboured, he thought the numbers very fair. Three stages were recognised, and it often happened that the patient had reached the third and worst stage before being admitted to the hospital; the percentage of those in the third stage worked out at 59 per cent. on all cases. It was not the fault of anybody connected with the hospital that the patients did not come earlier, but the fact remained that if the disease were arrested, that was all the doctors could be expected to do. More might be expected in a sanatorium, where three months was considered the ordinary time of residence for successful treatment, and three years was necessary for a cure—three years, that was to say, without a recurrence. Dr. Coghill had reported that while the leading continental sanatoria registered 11 per cent., 13·2 per cent., and 13·66 per cent. as really cured, Ventnor registered 17·14 per cent., and this was taking the average for 20 years. The committee endeavoured to be up to date; they kept in touch with what was going on at sanatoria at home and abroad. The patients had slept with open windows, and where possible out of doors, for some time past, and the other conditions required for the "open-air treatment" were attended to, *i.e.* fresh air, abundant food, and moderate exercise.

No New Method.

Dr. Robertson, one of the Ventnor physicians, maintained that the open-air method had been in force for as long as public opinion would permit. It was no new method, it was at least half a century old. The position of this hospital was one of evolution. Twenty years ago Sir Herman Weber, after expressing his delight at all he had seen there, said he should like to put all the beds in the balconies. At that time a very serious complaint was made about a patient leaning from the window on a winter's day—he assured the audience that a very serious complaint was made about it, and it was considered a fortunate thing that no harm resulted from it. The public was not prepared for the notion of open air for consumptives. Twenty years ago this hospital supplied pure air more liberally than any other hospital, 20 years ago the patients were not only being taught to eat, but to swallow an indiarubber tube to help them to take larger quantities. But the "open-air method" had been adapted at Ventnor, and it was being carried out. He looked forward to the time when it would be considered a reproach to allow consumptive patients to reach the third stage, and still to be living among their friends without a helping hand stretched out to ameliorate their condition.

Courage to Remodel.

The report and accounts were adopted; and, some re-appointments having been made, Lord Alverstone proposed a vote of thanks to Lord Rosebery. In the course of his remarks he alluded to three special points. Many years ago, a remark of Lord Randolph Churchill's, about sending consumptives to South Africa, led him (Lord Alverstone) to look into matters at Ventnor. He found that in many cases the lives of breadwinners of families were being saved—of both men and women—and if these lives were not saved, they were at any rate prolonged; this proved what a really charitable work was being done by this hospital. Secondly, he hoped that the governors would have the courage to re-model the system of admission so that the long waiting time might be done away with. If possible, a patient ought to be admitted within three weeks of the date of his acceptance by the doctor. Thirdly, he hoped that those ladies and gentlemen who had votes would endeavour to see that they were given to patients who would benefit by the treatment; it was not a hospital for hopeless cases, and it was not a home for the dying. The committee had found themselves seriously hampered by the fact that recommendations were given to hopeless cases, and he thought the governors should ascertain that there was a fair and reasonable expectation that benefit would result from the treatment before giving their votes.

